# Phosphorescence of the Human Body

**Published:** 1847-01

**Authors:** 


*Phosphorescence of the Human Body.*—The subject of this case was a male infant sixteen months old. The child had suffered from teething, and had been casually seen by Dr. H. M'Cormack, of Belfast. An emetic was administered, and an irritating liniment rubbed on the breast. The nurse, in raising the child in bed at night, observed a phosphorescent light about the hips, both before and after the candle had been lighted. The legs were also observed to be luminous for a short time. From what Dr. M'Cormack could learn, the appearances very much resembled that produced by phosphorized oil, but none of this had been employed. The phenomenon occurred only once. The mother had, however, observed, that on one occasion a spark (electrical) had flown to her hand from the infant's body. Cases of human phosphorescence in the living body are rare, and the fact recorded by Dr. M'Cormack is, therefore, interesting.—*Ib.*

